# Blue plaque review series: Mabel Purefoy FitzGerald (1872–1973)

**DOI:** 10.1113/EP092275

**Published:** 2025-03-07

**Authors:** Martha Tissot van Patot

**Keywords:** altitude, digestion, history of medicine, hypoxia, physiology, respiration, women

## Abstract

Mabel Purefoy FitzGerald (1872–1973) was the first centenarian to receive an honorary degree from the University of Oxford. She received an honorary bachelor of the arts 68 years after being the first woman to complete the Honour School of Physiology. Her work from a solo trip through the wild and unruly mining communities high in the Colorado Rocky Mountains in 1911 revealed that to compensate for the hypoxia of high altitude, residents had lowered CO_2_ and elevated haemoglobin. These data are some of the first to suggest a hypoxia‐sensing mechanism. Until recently, her other numerous achievements and astonishing experiences struggling to become a physician at the turn of the 20th century have remained lost to history. FitzGerald's numerous accomplishments are a testament to her passion for medical science and unparalleled courage in the face of innumerable setbacks.

## Abstract (highlights)

- **What is the topic of this review?**
  Until recently, the only thing Mabel Purefoy FitzGerald was recognized for was her research demonstrating that CO_2_ is reduced and hemoglobin increased as the altitude of residence is increased. Since then, extensive research into her archive and related documents has revealed that her historic contributions to science were far more extensive.
- **What advances does it highlight?**
  Historic documents reveal that FitzGerald was the first woman to study medicine at Oxford University, the first to publish evidence of circulatory compensation to protect oxygen supply in various cardiopulmonary diseases, the first to prove the origin of hydrochloric acid in the parietal cells of the stomach, and the first to show physiological differences between men and women in response to hypoxia.

## 1 INTRODUCTION

**FIGURE 1:**
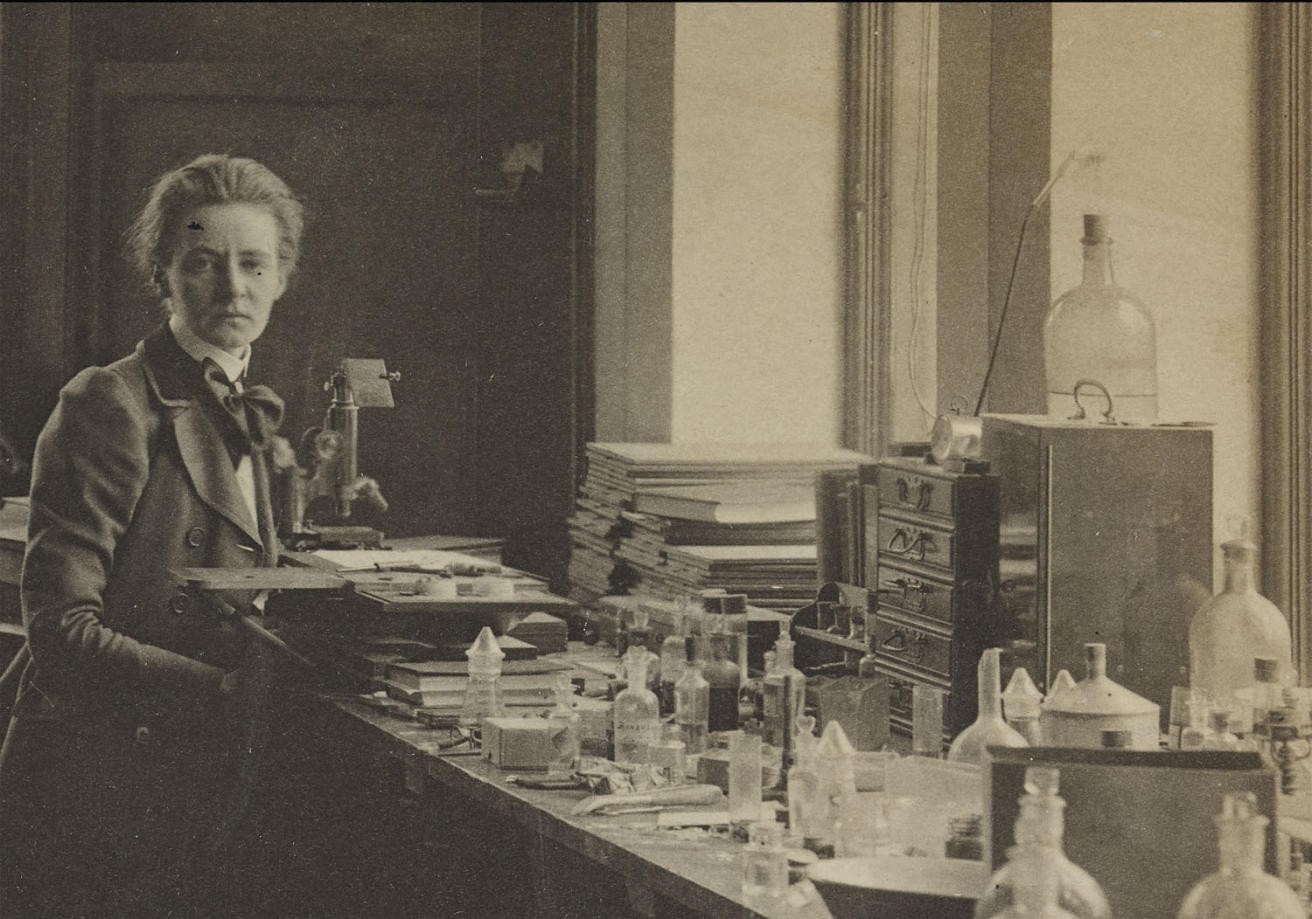
Mabel Purefoy FitzGerald (1872–1973) at the University of Copenhagen, Denmark, 1901. Photo courtesy of the Bodleian Library, University of Oxford, UK.

In 1913, Mabel Purefoy FitzGerald (Figure 1, 1872–1973) published an article titled ‘The changes in the breathing and the blood at various high altitudes’ (Fitzgerald, 1913). On her pioneering solo travels through the wild and unruly mining camps of the Colorado Rocky Mountains, she was the first to demonstrate that carbon dioxide is lowered and haemoglobin raised as the altitude at which people live increases. This unique research was later acknowledged upon his acceptance of the Nobel Prize in Physiology or Medicine in 2019 by Peter Ratcliffe, who lauded FitzGerald's data as some of the first to hint at a hypoxia‐sensing system.

Although FitzGerald pursued a career in medical science at the turn of the 20th century, little was known about her until the turn of the 21st century. Gabrielle K. Savard presented a brief overview of FitzGerald's life in 1997, at the International Hypoxia Symposium, and shortly thereafter, John West, Robert W. Torrance and John T. Reeves published brief histories of her life (Reeves & Grover, 2001; Savard, 1997; Torrance, 1999; West, 1998). These short biographies resulted from brief forays into the extensive archive of FitzGerald's personal and scientific papers, which she had donated to the Bodleian Library upon her death. A more extensive and detailed investigation of the 40 boxes overflowing with documents revealed an extraordinary set of circumstances under which FitzGerald sought an education, conducted research and pursued a career in medicine. Her resilience in the face of adversity is truly inspiring.

## 2 EARLY YEARS

Mabel FitzGerald was the last of seven children born to the FitzGerald family of North Hall Farm and Brickworks in Preston Candover, Hampshire, England, on 3 August 1872. Her mother noted the occasion in her diary simply as, ‘No. 8 born (Mabel Purefoy) at 8 A.M.’ (FitzGerald, 1882–1901; either she was so exhausted that she miscounted, or she had an unrecorded stillbirth or miscarriage). The last of Richard Purefoy FitzGerald and Henrietta Mary FitzGerald's (née Chester) brood would prove to be the most vivacious and energetic of the lot, always looking for partners to play tennis or snob cricket, host a musical evening, put on a play, or go for hours‐long adventures in the woods.

According to family diaries, the FitzGeralds were a close clan who enjoyed each other's company and whose home was often filled with visiting friends and family (FitzGerald, 1882–1901). Well into their twenties, the FitzGerald sisters spent their days accompanying their mother in doing good works, helping in the local church, and socializing with friends and neighbours. The women expected to continue in this fashion well into their dotage, the marriage market among the landed gentry being so small as to be non‐existent. However, in 1895, when Mabel was 22, both of her parents unexpectedly died of acute illness within months of each other.

The sisters went to stay with their paternal grandmother at Shalstone Manor Farm to recover from the shock and decide their futures. Their grandmother, Sara Anna Elizabeth FitzGerald (née Jervoise), was an unusual woman. She had run her own estate with great success since the death of her husband decades earlier. Her friends and confidants included some of the era's greatest poets, politicians and artists. Eliza, as she was known, held a strong belief in the education of women and was closely involved with her granddaughters’ education. Robert Browning dubbed her the Learned Lady (Browning, 1966).

## 3 STARTING THE SLIDE INTO MEDICINE

Eliza recognized something special in her youngest granddaughter and asked the local physician, Dr George Hanby De'ath, to encourage Mabel toward district nursing. Although fascinated by local health lectures on digestion and the heart, FitzGerald was not enamoured with the idea of cleaning up bodily fluids, changing and washing sheets and taking orders. When her sisters decided to move into a home in Oxford together and continue the life they had always known, FitzGerald surprised everyone by joining them. In October 1896, within weeks of moving to Oxford, she enrolled in chemistry and zoology classes through the Association for the Education of Women, marking the beginning of her challenging journey into the world of medicine (FitzGerald, 1914–1973).

She was enthralled. Each night, she carefully copied her notes and drawings into bound books, committing every tiny detail to memory. She continued through the spring semester, becoming more and more drawn to the study of medicine.

In England, the spring of 1897 brought not only rain and flowers but riots protesting the presence of women in higher education. An effigy of a woman riding a bicycle wearing blue stockings was burned in a riot at the University of Cambridge. The figure represented women with the audacity to want to learn as much as men, who were derogatorily referred to as bluestockings. Dire warnings filled the newspapers that women would go mad, become infertile and even fall ill and die if they attempted to fill their heads with ideas above their intelligence.

## 4 FIRST WOMAN IN MEDICINE AT THE UNIVERSITY OF OXFORD

Amid all this chaos, FitzGerald walked into the office of Francis Gotch, the Waynefleet Professor of Physiology, and asked permission for her and a friend, Grace Devitt, to attend the Final Honour School of Physiology. Gotch allowed FitzGerald and Devitt to attend the programme unofficially. Thus, Mabel Purefoy FitzGerald and Grace Devitt were the first women to study medicine at the University of Oxford. Unfortunately, as the daughter of a produce broker, Grace's money to attend university ran out after the first year and she left to become a nurse at the Reading Infirmary.

The FitzGerald family estate provided each daughter with a moderate income. Their grandmother taught them how to save and invest their money. Mabel was reasonably successful, and although money was often tight, she was able to continue with her education.

The Honour School of Physiology was the equivalent of the medical school at Oxford at the time. Upon completion of exams, male students earned a Bachelor of Medicine degree. With that degree, they could train in London hospitals before taking examinations to obtain their physician's licence. Because Oxford would not award degrees to women, this option was not available to FitzGerald. However, Gotch later said that FitzGerald's performance was one of the primary reasons he eventually opened the Honour School to women.

### 4.1 RESEARCH YEARS

Impressed with FitzGerald's performance in the first year of the honour programme, lecturer Gustav Mann, who was putting together his epic tome on histological methods (Mann, 1902), asked FitzGerald to work in his laboratory. In 1899, after a year of working in his laboratory, Mann and future Nobel Laureate Charles Sherrington gave FitzGerald an independent research project detailing the anatomy of the macaque spine (FitzGerald, 1906). Through her hard work, she developed an expertise in physiological histology.

FitzGerald's work with Mann and Sherrington continued through her time at the Honour School of Physiology. In the midst of her studies and research, an opportunity arose for her to spend 6 months learning bacteriology from Carl Julius Salomonsen (considered the Father of Bacteriology in Denmark) at the University of Copenhagen. While there, she and his former pupil Georges Dreyer (who became the first Chair of Pathology at the University of Oxford in 1907) conducted a research project to improve upon a method to identify water sources contaminated with typhoid (FitzGerald & Dreyer, 1902). In 1902, she was invited to present their work at the opening of the Statens Serum Institut to a room full of future Nobel Laureates and groundbreaking scientists, including Svante Arrhenius, the first to predict global warming from the use of fossil fuels; Robert Koch, who discovered the tuberculosis bacterium; Paul Ehrlich, an immunologist who found a cure for syphilis and coined the term chemotherapy; and Gerhard Armauer Hansen, who discovered the cause of leprosy (Hardy, 2010).

FitzGerald completed the Honour School in 1904, yet without a degree she could not pursue her dream of becoming a physician. Refusing to give up on her passion for medicine, she decided to pursue a career in research. Because women were not granted faculty positions, laboratories or funding, the only way she could proceed was to accept opportunities to work in the laboratories of her mentors and their colleagues. Thus, FitzGerald began a 14‐year career in research that took her to four countries and produced 11 publications.

In 1905, after completing the Honour School, FitzGerald accepted an invitation from respiratory physiologist John Scott Haldane to determine normal CO_2_ values and how they change in various cardiopulmonary and vascular diseases (Fitzgerald, 1910a; Fitzgerald & Haldane, 1905). She arranged to meet with the new Regius Professor of Medicine, William Osler (known as the Father of Modern Medicine), to obtain permission to measure CO_2_ in patients at the Radcliffe Infirmary. Impressed with the young woman on learning her story, Osler invited her to attend the clinical teaching course he was initiating, making FitzGerald the first woman to study clinical medicine at Oxford.

While learning from Osler and doing research with Haldane, she also developed an improved method to diagnose ringworm. She did a project with T. S. P. Strangeways at Cambridge, bringing the opsonic index method into serious question (Fitzgerald, 1908; FitzGerald et al., 1907). Their work also produced a more reliable method for counting cells, the principle of which remains in use over 100 years later. Her drive to learn as much as possible led her to study clinical pathology with James Ritchie (who held the first pathology appointment at the University of Oxford and was a founder of the Pathological Society of Great Britain and Ireland and the *Journal of Pathology and Bacteriology*). She assisted him and Osler in establishing the first in‐hospital clinical pathology laboratory at the Radcliffe and worked in the lab, all while continuing her studies with Osler and various research endeavours. FitzGerald was happiest when busy.

She had completed three years of clinical training and had studied for the equivalent of a bachelor degree in medicine, yet with no degree she still had no way of becoming a doctor. Therefore, in 1909, she accepted a fellowship at the newly established Rockefeller Institute in New York. The transition across the Atlantic marked a new and less salubrious chapter in her life.

The institute director, Simon Flexner, assigned Hideyo Noguchi (a controversial scientist whose work in syphilis and yellow fever proved difficult to repeat by other investigators) as a mentor to FitzGerald. He was a secretive and difficult person with arguably less medical education and research experience than FitzGerald. He assigned her the menial task of optimizing cell culture conditions for bacterial sporulation. She and another fellow, Maud Menten (who published the Michaelis–Menten equation describing enzyme kinetics in 1913), bonded over their disgust at the appalling treatment of women at the institute. For example, women were prohibited from dining with men at lunch, leaving them out of all scientific discussions outside formal laboratory meetings.

FitzGerald finished her assigned project early and arranged to accompany Menten to the University of Toronto to complete her fellowship under Menten's former advisor, A. B. Macallum (the founder of the National Research Council of Canada and the first to chair the Biochemistry Department at the University of Toronto), while Menten pursued her medical training. For the past 50 years, Macallum and many other investigators had been trying to improve Claude Bernard's method for identifying where acid originates in the stomach (Bernard, 1859; Brücke, 1859; Hallett, 1871; Heidenhain, 1870). He warned FitzGerald that she would be unlikely to accomplish much in the short time (3 months) remaining on her fellowship award. Using her considerable expertise in histochemical staining methods, FitzGerald solved the methodology problem and was the first to show that hydrochloric acid originates in the parietal cells (FitzGerald, 1910b, 1910c). She never received credit for her work due to the jealousy of other researchers (Tissot van Patot, 2021).

## 5 IN PURSUIT OF A MEDICAL DEGREE

In the spring of 1910, FitzGerald returned to New York. Because women were permitted to earn medical degrees in the United States, she decided to finally earn her medical credentials and applied to Cornell University Medical School. Her timing was terrible. The American Medical Association (AMA) and the Carnegie Foundation had recently embarked on a plan, as advocated by the Flexner report (Flexner, 1910), to force the closure of medical schools they deemed unacceptable and develop much more rigorous standards for those entering medical school (Weiss & Miller, 2010). There is some controversy about the intent of the AMA and Carnegie. Regardless of intent, medical schools closed in record numbers, and the ranks of women and minorities in medicine plummeted (Barkin et al., 2010; Joseph et al., 2021; Weiss & Miller, 2010). This was because many of the schools that closed admitted large numbers of women and minorities and because the rigorous entry requirements were most easily met by privileged white men.

FitzGerald's application included her extensive credentials, which included more than 1700 h of medical education, nine scientific publications and 3 years of clinical training with Sir William Osler. Cornell denied her application on two points. She needed more classes in biological chemistry (she already had the requisite hours and publications showing her expertise in this field) and physics (she had no physics hours). Also, the University of New York Board of Regents required people applying to medical school first to obtain a medical student certificate. Applicants received a medical student certificate by demonstrating they had a high school degree. Because she was educated by a governess, FitzGerald did not have a high school degree. The Regents required her to go to an American high school.

Over the next 2 years, FitzGerald attended Senftner Preparatory Academy in Manhattan while simultaneously taking physics at New York University and biological chemistry as a graduate student at Columbia University. She also took a brief trip to Oxford to visit family, during which time she attended additional chemistry and physics courses at the university.

In 1911, on her first summer break from high school, FitzGerald was invited to join Haldane's Anglo‐American Pikes Peak Expedition to the Colorado Rockies to explore how people adapt to hypoxia at high altitudes. Rather than join the men at the top of the peak, she travelled alone throughout remote and wild mining towns, measuring CO_2_ and haemoglobin in the men and women residing there. When she returned to high school in the autumn, in addition to studying, she wrote a manuscript that earned recognition more than 100 years later as an influential paper in unravelling the hypoxia sensing system (FitzGerald, 1913; Ratcliffe, 2019).

After completing her high school requirement, FitzGerald again applied to Cornell and the Regents, only to be told the rules had changed and she needed to attend college for 1 year. Because Oxford did not award her a degree, her work there was not recognized as adequate. She enrolled at New York University. Upon completing her studies, she again applied for her medical student certificate, only to be informed that the University of New York Board of Regents did not recognize New York University's credentials, and her entire application was summarily dismissed.

### 5.1 FINALLY, A DOCTOR?

Within days of this devastating rejection, FitzGerald received a telegram from her former clinical pathology mentor, James Ritchie, asking her to come to Edinburgh to replace one of his clinical pathologists leaving to join the war effort. It was the spring of 1915, and the First World War was well underway. So, FitzGerald, deemed unfit for medical school, moved to Scotland to work as a clinical pathologist at the Royal Infirmary of Edinburgh. Much of her time was spent in the lab, but she also attended patients’ bedsides doing physical exams, taking histories and collecting samples to help with diagnoses.

She resumed her quest to become a doctor by applying to the Royal College of Physicians and Surgeons of London. Seeing no irony in suggesting that she would be too busy working as a clinical pathologist to earn her medical licence, the college wrote that if she were determined to move forward, she would first have to attend pharmacology and more anatomy classes before taking their examinations. At night, after working as a physician all day, FitzGerald studied pharmacology and anatomy, which she had already taken at Oxford. When she submitted these courses to the college, they accepted her credentials for her pharmacology course at the University of Edinburgh but refused to recognize her anatomy courses from the Royal College of Surgeons of Edinburgh because the Royal College of Physicians and Surgeons of London did not acknowledge the authority of the Royal College of Surgeons of Edinburgh.

Throughout her years of struggle to become a physician, FitzGerald does not share her feelings about the matter with anyone, not even her beloved sisters. She expresses frustration with Simon Flexner, Hideyo Noguchi and over a falling out with Georges Dreyer but keeps her feelings about her pursuit of a medical degree to herself. One can only speculate the gut‐wrenching feelings of frustration, anger and hopelessness that must have assaulted her every time a new correspondence arrived, denying her achievements and preventing her from pursuing her dream.

### 5.2 THE END OF THE BATTLE

At the end of the war, the clinician she replaced returned, and despite her colleagues pleading with the infirmary board to retain her services, FitzGerald lost her job. She obtained a position teaching bacteriology for the Royal College of Surgeons of Edinburgh and continued taking courses and examinations toward earning her medical licence into the early 1930s. Finally, in her 60s, she was forced to give up her dream of becoming a doctor and return to Oxford to care for her beloved ageing sisters.

Long after her sisters passed away, her neighbour and friend Sir Roger Elliot (a British theoretical physicist and a pioneer in the use of quantum theory in solid state physics), applied to Oxford to get FitzGerald the recognition she was due. In 1972, this intrepid, persistent, brave woman became the first centenarian awarded an honorary degree by the University of Oxford an honorary bachelor of the arts.

By delving into women's lives in the history of medicine, we discover that their roles in history go far beyond the one or two discoveries for which they are noted. Careful investigation of FitzGerald's life has revealed new historical information and a greater understanding of challenges faced by women at the turn of the 20th century. The lives of many more women in scientific history are ripe for discovery, and the discipline is primed to prosper from the knowledge.

## AUTHOR CONTRIBUTIONS

Sole author.

## CONFLICT OF INTEREST

None declared.
